# Social origin, field of study and graduates’ career progression: does social inequality vary across fields?^1^

**DOI:** 10.1111/1468-4446.12696

**Published:** 2019-08-14

**Authors:** Marita Jacob, Markus Klein

**Affiliations:** ^1^ University of Cologne; ^2^ University of Strathclyde School of Education

**Keywords:** social origin, field of study, occupational prestige, career, social inequality

## Abstract

Research on stratification and mobility has consistently shown that in the UK there is a direct impact of social origin on occupational destination net of educational attainment even for degree‐holders. However, only a few studies applied a longitudinal and dynamic perspective on how intergenerational mobility shapes graduates’ working careers. Using multilevel growth curve modelling and data from the 1970 British cohort study (BCS70), we contribute to this research by looking at the emergence of social inequalities during the first ten years since labour market entry. We further distinguish between graduates of different fields of study as we expect social disparities to develop differently due to differences in initial occupational placement and upward mobility processes. We find that parental class does not affect occupational prestige over and above prior achievement. Separate analyses by the field of study show that initial differences in occupational prestige and career progression do not differ between graduates from different classes of origin in STEM fields, and arts and humanities. It is only in the social sciences that working‐class graduates start with lower occupational prestige but soon catch up with their peers from higher classes. Overall, our results indicate no direct effect of social origin on occupational attainment for degree‐holders once we broaden our focus to a dynamic life course perspective.

## Introduction

Numerous studies in research in social stratification and mobility have found that social origin and educational attainment are both strong determinants of occupational attainment. The Blau–Duncan model (1967) already addressed life‐cycle variations in occupational attainment, but scholars have used dynamic approaches to modelling intergenerational inequality in occupational careers only recently. In such a life course perspective, one asks whether initial differences in occupational attainment between individuals from different social origin and education groups perpetuate or increase over the life course or whether career developments offer the potential to compensate for initial differences (e.g., Mayer 2009; Sørensen 1975). A dynamic perspective on career advancement and progression is strongly intertwined with current conceptual advances in theories of cumulative (dis‐) advantages (e.g., DiPrete and Eirich 2006).

To account for inter‐ and intra‐generational social mobility a few recent studies have examined occupational trajectories and career progression of individuals with different social and educational backgrounds (e.g., Barone, Lucchini and Schizzerotto 2011; Härkönen and Bihagen 2011; Manzoni, Härkönen and Mayer 2014; Passaretta, Barbieri, Wolbers and Visser 2018; Schulz and Maas 2012). Whereas this literature documented differences in career advancement *between* these groups and over time, we contribute to previous research by looking at *within*‐group differences in occupational careers among *degree‐holders.* It allows us to evaluate the impact of so‐called ‘qualitative’ differences that are assumed to be increasingly important as more and more individuals attain higher education in the course of educational expansion (e.g., Lucas 2001). A growing literature in education, as well as stratification research, is concerned with graduates’ field of study as an essential dimension for variation in labour market outcomes (e.g., Klein 2011; Van de Werfhorst 2002).

Several studies provide empirical evidence for differences between graduates from different fields of study in various labour market returns such as occupational prestige (Katz‐Gerro and Yaish 2003; Shwed and Shavit 2006), class position (Sullivan et al. 2018a) or earnings (Kim, Tamborini and Sakamoto 2015; Laurison and Friedman 2016; Sullivan et al. 2018b). Existing research commonly uses ‘snapshot’ measures at labour market entry or a particular age. However, occupational careers of graduates from different fields of study may evolve differently with some fields offering better chances of occupational upward mobility than others (cf. Rosenfeld 1992). In consequence, initial social inequalities in labour market outcomes might decrease over time in some fields, whereas in other fields social inequalities might even aggravate in the course of individuals’ career progression. Using growth curve modelling allows us to describe occupational stratification across the life course by simultaneously taking into account inequality at labour market entry, the rate of career progression and the length of career progression (Manzoni et al. 2014).

In our article, we therefore analyse occupational trajectories by family background within graduates from different fields of study. The innovative contribution of the paper is threefold: first, our *within‐group comparison* allows us to analyse the impact of social origin on career trajectories among the highest qualified that remains unobserved when modelling social inequalities across the life course and controlling for educational attainment (e.g., Manzoni et al. 2014). Second, we consider the heterogeneous effects of social origin on intragenerational mobility by analysing social disparities in *field‐specific careers*. Third, by observing a time‐span of 10 years since the labour market entry, we provide evidence for a potential *long‐term impact of social origin* in the course of individuals’ working careers. For our empirical analyses, we use data from the 1970 British Cohort Study and growth curve models to describe within‐ and between‐individual occupational trajectories holistically.

## Previous research: social origin, field of study and labour market returns

### Family background and graduate’s career progression

Since the seminal book of Blau and Duncan (1967), research in social stratification and mobility asks whether and to what extent social origin affects the labour market entry and later stages in the working life. The Blau–Duncan model already emphasized the importance of dynamic modelling and career progression for intergenerational mobility processes. However, many studies applied cross‐sectional analyses to examine the direct effect of social origin on (graduates’) labour market destination using population samples (e.g., Laurison and Friedman 2016; Wakeling and Savage 2015), considered outcomes at particular points in time during the career (Britton, Shephard, Vignoles and Dearden 2016; Crawford and Vignoles 2014; Macmillan, Tyler and Vignoles 2015) or at specific ages (Crawford et al. 2016; Gugushvili, Bukodi and Goldthorpe 2017; Sullivan et al. 2018a, 2018b).

Jacob, Klein and Iannelli (2015) and Bukodi and Goldthorpe (2011) go beyond these single observations of labour market outcomes, but still, use only two‐points‐in‐time measures of individual careers. Both studies found initial social disparities in graduates’ labour market outcomes that decreased across the working life in the UK. Based on REFLEX data, Jacob et al. (2015) showed that parental education has a positive effect on accessing the higher salariat class at labour market entry, but this result does not hold five years after graduation. Using three British cohort studies (1946, 1958, 1970), Bukodi and Goldthorpe (2011) examined individuals’ occupational earnings. Among degree‐holders, they identified social inequalities in occupational earnings in the first job but a smaller social gradient in the mid‐thirties at the time of ‘occupational maturity’.

In recent years, social stratification scholars have used new approaches of dynamic statistical modelling, that is, multilevel growth curve models, to account for social origin differences in career advancement (e.g., Barone, Lucchini and Schizzerotto 2011; Härkönen and Bihagen 2011; Manzoni et al. 2014; Passaretta et al. 2018; Schulz and Maas 2012). However, all of these studies consider the whole working population without modelling interacting effects between social origin and educational attainment on career development. Also, previous research did not investigate more fine‐grained social gradients within the same level of education.

### Graduates’ field of study and career progression

The field of study is a significant determinant of graduates’ labour market outcomes in the UK. Reimer, Noelke and Kucel (2008) showed that occupational status varies considerably between graduates from different fields. In particular, graduates from education and technical fields obtained a socioeconomic status above average, whereas degree‐holders in health and welfare and agricultural fields had the least favourable positions. In contrast, Kim and Kim (2003) found that graduates from education and humanities and arts are disadvantaged in access to the upper service class compared to graduates in health or engineering. Regarding financial returns, Chevalier (2011) showed substantial heterogeneity in the average wages of UK graduates from different fields 3.5 years after graduation. Graduates from medicine, architecture, and engineering have the highest wage returns; graduates from linguistics, communication, and arts have the lowest. Bratti, Naylor and Smith (2008) considered wage returns of UK graduates from different fields at age 30 and showed that graduates from the social sciences had the highest wage returns, graduates from the sciences were in the medium position, and graduates from the arts and humanities had the lowest wage returns among all fields (cf. Walker and Zhu 2011).

Only a few studies so far devoted attention to field‐specific career advancement, and if they did, they concentrated on earnings and in most cases were based in the US. Thomas and Zhang (2005) compared the initial wages of graduates to those achieved four years after graduation. They observed widening initial earnings differences between graduates from business, math, science, social sciences, and graduates from education‐related fields and history. Graduates from health and engineering started their career with high initial earnings but had a rather small increase across the four years after graduation. Finnie and Frenette (2003) analysed the earnings growth of Canadian graduates two and five years after graduation. They showed that (male) graduates from arts and humanities have the highest increase in average earnings over time, but still lag behind the earnings of their peers graduating in other fields. Using data of a panel study combined with administrative tax data, Kim, Tamborini and Sakamoto (2015) found that STEM (science, technology, engineering and maths) graduates have significant advantages regarding lifetime earnings gains compared to graduates from the social sciences.

Studies addressing variation between fields of study in social gradients in labour market returns are rare and often limited to wages and income. In a Norwegian study, Hansen (2001) showed that social inequalities in income are smaller for graduates in STEM fields than for graduates in humanities and arts. For the Appalachian region in the US, Wolniak, Seifert, Reed and Pascarella (2008) found that pre‐college family income is significantly associated with earnings for all fields except technical and applied fields. It is the greatest for STEM fields, smallest for education fields and health sciences with arts and humanities in between. A recent study in the US by Manzoni and Streib (2018) showed no differences by parental education in earnings ten years after graduation for graduates in STEM majors but identified an earnings advantage for male graduates in arts and humanities with highly educated parents over their peers from less‐educated families.

Similarly, Hällsten (2013) found that the effect of the class of origin on wages in the Swedish labour market is more substantial for graduates from humanities and arts than for graduates from the sciences. For the UK, Macmillan, Tyler and Vignoles (2015) considered variation in social gradients in access to higher managerial positions between different labour market sectors and found a positive association between parental class and entering a business, medical or law profession 3.5 years after graduation. None of these studies model variation in social inequalities across fields of study in career trajectories across the life course.

Summing up, we identified the following gaps in the literature: First, the few given ‘snapshot’ studies of graduates in the UK lacked a profound continuous empirical measurement of career trajectories and did not sufficiently answer how social inequalities develop across careers. Second, many studies were concerned with field‐specific wages and earnings at labour market entry but did not consider field‐specific career progression in occupations that may reveal different patterns. Finally, only a few studies investigated social inequalities in labour market outcomes by field of study and, if so, did not consider career progression over time. However, the average effect of social origin on career trajectories may be misleading if social inequalities may decrease or increase across the working life in different fields.

## Theoretical considerations: social origin and field‐specific careers

To explain social gradients in labour market outcomes among individuals with the same education, previous literature on intergenerational mobility commonly refers to parents’ economic, social and cultural capital that they transmit to their children (Bourdieu 1986; Coleman 1988). Parents’ economic resources are expected to exert an influence on their offspring’s labour market outcomes by, for instance, facilitating job search or residential mobility. Parents’ social networks may provide access to information about vacancies or potential employers. Cultural capital is considered to be beneficial in the recruitment process (cf. several contributions in Bernardi and Ballarino 2016).

Regarding career development after graduation, we need to distinguish between (1) initial labour market attainment directly after graduation (differences in intercepts), and (2) career progression over time (differences in slopes). We assume parental resources and support to be particularly helpful at labour market entry when employers hire applicants for the first time, and signals of potential productivity are limited. Later in the career, employers can either observe employees’ productivity directly or evaluate job applicants’ accumulated work experience. Graduates from the lower class of origin may, therefore, compensate for initial disadvantages and catch up with their peers from the higher class of origin across the career. We expect that there are social class differentials at labour market entry. However, these class differentials vanish across the career due to the steeper career progression of those from the lower class of origin (*Hypothesis 1*). For the UK, empirical results by Bukodi and Goldthorpe (2011) and Jacob et al. (2015) already provide some support for the assumption of convergence between graduates’ careers.

To derive hypotheses on field‐specific social class differentials in career advancement, we use a combination of theories on returns to field‐specific skills, labour market allocation and promotion processes, and different labour market segments. First, a standard explanation for varying returns to fields of study is differences in the *type of graduate skills* (Heijke, Meng and Ramaekers 2003; Paglin and Rufolo 1990; Van de Werfhorst and Kraaykamp 2001). For example, Van de Werfhorst and Kraaykamp (2001) distinguish four types of endowments: cultural, economic, communicative, and technical resources. Graduates with high levels of economic skills (e.g., business and administration, law) and technical skills (e.g., technical fields, maths and sciences) achieve more rewarding occupational positions in the labour market whereas graduating in fields linked to cultural skills (e.g., arts and humanities, education) yields the lowest labour market returns. However, these considerations refer to average returns in the labour market and do not imply any expectations about career progression. Assuming ceiling effects, graduates starting in high‐status positions at labour market entry may have fewer chances for improvement.

Second, Klein (2011) argues that employers have imperfect information about labour market entrants’ productivity and training costs and, therefore, graduates from fields providing ‘soft skills’ have only a weak signalling value due to their low occupational specificity. As tenure and work experience grows over time, graduates from unspecific fields can correct initial mismatches, move ahead in their careers and gain promotions as employers can judge them more accurately. Over‐education is less likely to occur among labour market entrants from ‘occupation‐specific’ fields, and therefore, there is less need for later corrections.

Finally, the third line of theoretical explanations for differences between fields of study in career progression focuses on *characteristics of graduates’ jobs* (e.g., Giesecke and Schindler 2008)*.* This approach distinguishes between highly regulated labour market sectors with fewer opportunities (and less necessity) for occupational and career mobility and flexible market segments with high rates of temporary employment. Strong linkages between fields of study and occupational labour markets may, therefore, be advantageous for entry into high‐status occupations and long‐term occupational stability. Summing up, we can expect appropriate high‐status positions at labour market entry for graduates from occupation‐specific fields, such as STEM fields, and less intragenerational mobility over time compared to graduates in social sciences, arts, and humanities.

Taking into account social origin, we assume parental resources to compensate for signalling of unspecific skills and parental resources to be particularly crucial at labour market entry. Hence, for graduates from the arts and humanities with the least specific skills, we expect significant social inequalities in initial labour market allocation. However, it is not clear whether and how social gradients develop across the career of graduates from arts and humanities. On the one hand, students from lower classes of origin may overcome their initial disadvantages and catch up with those from a higher class of origin as their real productivity becomes visible throughout their career (*Hypothesis 2a*). On the other hand, if career advancement and promotion in the labour market of arts and humanities still requires social and cultural resources, the advantages of offspring from higher classes may persist or become more significant due to cumulative advantages (*Hypothesis 2b*).

Graduates from STEM fields have acquired more specific skills and qualifications compared to graduates from other fields. Thus, in the matching occupational labour market, parents from STEM graduates should have less leeway on job allocation procedures than in other partial labour markets. Also, we assume that STEM graduates enter occupations with relatively high task specificity and thus productivity is more dependent on what was taught during graduates’ studies than on pre‐existing skills or resources acquired in the parental home. Therefore, we expect social inequalities among STEM graduates to be small both in initial placement and career progression (*Hypothesis 3*).

**Figure 1:**
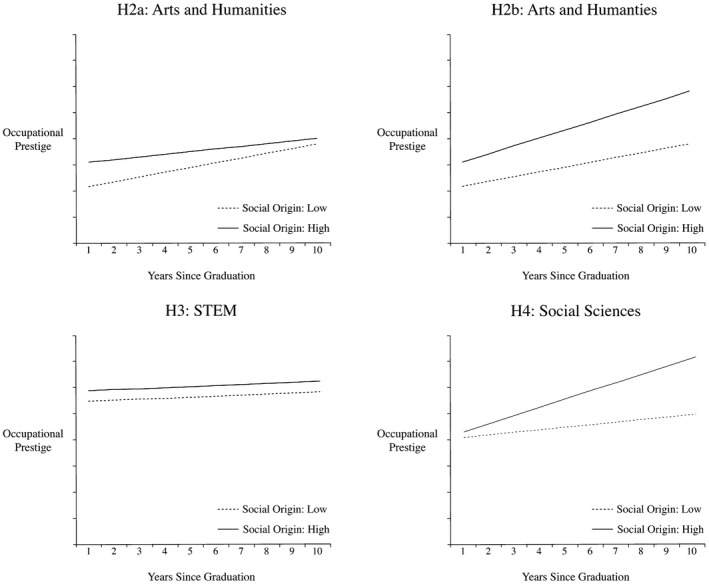
Schematic illustration of hypotheses 2a, 2b, 3 and 4: Graduates' career development by social origin in different fields of study

In the social sciences (including business and administration, economics), we expect initial inequalities and increasing social inequalities over time as social and cultural capital acquired in the parental home may facilitate access to and promotion in services and higher managerial positions (Jackson, Goldthorpe and Mills 2005). Also, for these graduates, the intergenerational inheritance of business and self‐employment may play a crucial role in career progression. Hence, we expect a divergence of occupational positions over time due to a steeper slope for those from higher classes (*Hypothesis 4*). Figure 1 illustrates these field‐specific hypotheses.

## Data, variables and methods

### 1970 British Cohort Study (BCS70)

For our empirical analysis, we use the 1970 British Cohort Study (BCS70) which follows the lives of people born in England, Scotland and Wales in a single week of 1970 (Elliott and Shepherd 2006). The original sample consisted of over 17,000 individuals. At the ages of 5, 10, and 16 cohort members were followed up by parental interview and examination. At the age of 26, cohort members filled in a postal questionnaire and were then interviewed at four‐year intervals from the age of 30 onwards. In our paper, we use information collected in the third sweep (age 10, Butler and Bynner 2016) and sweeps six to eight (age 30–38, University of London 2016a, 2016b, 2016c). We additionally draw on the 1970 British Cohort Study Activity Histories dataset (University of London 2016d) including retrospective information on activity histories from the age of 16 until the interview date of sweep 8, that is, until age 38 (Hancock et al. 2011). We use retrospective information on the field of study, type of degree, marital status and number of children from sweeps 6 to 8 (age 30–38), and identified individual background information on the parental class, cognitive ability and ethnicity in the third sweep (age 10).

As with other longitudinal studies, the BCS70 is subject to considerable unit non‐response across all waves. Only around 20 per cent of cohort members participated in all existing waves, over half of the cohort members dropped out of at least one wave but returned to the study and one third dropped from the study entirely (Mostafa and Wiggins 2015). Unit non‐response is not random, mainly due to residential moves (Elliott and Shepherd 2006), but the predictive power of birth characteristics for unit non‐response is consistently weak across waves and, therefore, undermines the efficacy of using non‐response weights (Cumming and Goldstein 2016; Mostafa and Wiggins 2015). There are changes in the parental class composition across waves, but the proportion of fathers with manual occupations was mainly falling between sweep 1 and sweep 3 and did not considerably change in later sweeps (Mostafa and Wiggins 2015).

Our analytical sample consists of university graduates (undergraduate and postgraduate) who studied full‐time and graduated from a UK HE institution until the age of 34. We observe graduates’ career progression from their first significant job (lasting at least six months) until up to 10 years in the labour market. We exclude part‐time students from our sample since their educational attainment runs in parallel to their occupational career, and they are likely to have a more stable starting point after graduation when compared to full‐time graduates. We identified 1,347 graduates at age 34 who were full‐time students, for whom information on the timing of graduation was available and who had at least one significant job during the observation period. After listwise deletion, our sample includes 951 complete cases with 99,399 person‐months.

### Variables and operationalization

Table 1 provides an overview of the variables and descriptive statistics for complete cases. Our dependent variable is the *Treiman occupational prestige scale* (SIOPS) measured at each job across the observation period (Treiman 1977). This internationally standardized scale of occupational prestige shows remarkable stability across time and countries and is widely used in life course analysis of occupational attainment (Härkönen and Bihagen 2011; Härkönen, Manzoni and Bihagen 2016; Manzoni, Härkönen and Mayer 2014). Occupational prestige is defined as ‘the general level of social standing enjoyed by the incumbents of an occupation’ (Hauser and Warren 1997, p. 188) and thus an essential measure of stratification.^2^ To assign SIOPS scores to occupations, we recoded British SOC90 occupational codes into ISCO‐88 codes. In our sample of graduates, SIOPS scores range between 15 (e.g., building construction labourers) and 78 (e.g., university professors and medical doctors), with an average of 54.79 and a standard deviation of 11.26. On average, we identify 1.22 changes in occupation/occupational prestige in our observation period. For 62 per cent of respondents, we observe at least one change in occupation/occupational prestige across the observation period.

**Table 1:** Summary statistics (*N* = 99,399 person‐months from 951 graduates)

	Mean/percentage	SD	Min	Max
SIOPS prestige score	54.29	11.26	15	78
Months since first significant job	58.68	34.52	1	120
CM female	0.49			
*Class of origin*				
Working class	0.12			
Intermediate class	0.31			
Salariate class	0.57			
Ethnic origin: non‐UK	0.04			
Cognitive ability	113.52	12.20	70.44	151.20
*Field of study*				
Humanities	0.30			
Social sciences (incl. professions)	0.32			
STEM fields	0.32			
Law and medicine	0.06			
CM with postgraduate degree	0.10			
*Marital status*				
Single	0.67			
Married	0.31			
Divorced	0.02			
*Number of children*				
Zero	0.86			
One	0.11			
Two	0.03			
Three or more	0.00			
CM in part‐time employment	0.07			

Note:

Statistics pertain to complete cases in our analytical sample. CM=Cohort member.

We measure *months since labour market entry* as the time since graduates started their first significant job, lasting at least six months. Since we intend to capture inequalities among graduates in career progression across the life course, we consider the time since individuals entered the labour market rather than their actual work experience. We follow graduates for up to ten years since their labour market entry, that is, the measure is right‐censored at 120 months. The average time that we observe graduates since labour market entry is around six years (SD = 34.52 months). To avoid the predefinition of a functional form and to allow for variation in career progression across different stages, we model career progression as five 24‐month splines and estimate linear slopes for each range.

Our measure of months since labour market entry does not account for career circumstances, career breaks, and re‐entry into the labour market due to family formation. Therefore, we control for *part‐time employment*, *the number of children,* and *marital status*. All these control variables are time‐varying measures to account for changes in family circumstances across the occupational career.

We operationalize our main independent variable, *class of origin,* with the 7‐class ‘analytical’ version of the National Statistics Socio‐Economic Classification (NS‐SEC), an improved operationalization of the EGP class schema (Goldthorpe 2007). To construct NS‐SEC codes, we rely on data on parents’ occupation provided by Gregg (2012). We use the highest class position of both parents and aggregate this measure into three classes differentiating between salariat class (1. higher and 2. lower managerial and professional occupations), intermediate class (3. intermediate occupations, 4. small employers and own account workers, 5. lower supervisory and technical occupations) and working class (6. semi‐routine and 7. routine occupations).

To operationalize *field of study*, we differentiate between three broad groups: (1) humanities (including arts), (2) social sciences (including economics and business), and (3) fields in STEM (science, technology, engineering, and mathematics) (for details see Appendix Table A.I).^3^ Field of study is operationalized as time‐varying since individuals may change their field of study from undergraduate to postgraduate studies or may study for a second degree in a different field. The *type of degree* differentiates between first degree and postgraduate degree. Likewise*,* we measure this in a time‐varying way so that it captures changes in educational attainment.

We also control for background characteristics such as *gender* and *ethnic origin*. Due to the small sample size, we only differentiate between cohort members originating from the UK and those originating from elsewhere.

Following Breen and Goldthorpe (2001), we operationalize *cognitive ability* by totalling all four conducted British Ability Scales (BAS) assessments (Elliott, Murray and Pearson 1979) – two verbal subscales (word definitions and word similarities) and two non‐verbal subscales (recall of digits and matrices) – and standardized it to a mean of 100 and a standard deviation of 15.

### Analytic strategy

To analyse the association between graduates’ social origin and occupational attainment over the early working life, we use growth curve modelling (Halaby 2003; Steele 2008). Growth curve modelling allows for the inclusion of time‐constant and time‐varying variables while putting particular emphasis on the modelling of time, in our case, career progression over the early life course and its association with covariates (i.e., differences in career progression by covariates).

Our baseline growth curve model is as follows:

$$\begin{array}{c} y_{\mathrm{it}}=\beta_{0}+\sum_{s=1}^{5}\beta_{1s}Time_{\mathrm{its}}+\beta_{2}Class_{i}+\beta_{3}Ethnic_{i}+\beta_{4}Cog_{i}+\beta_{5}Field_{\mathrm{it}}+\beta_{6}Degree_{\mathrm{it}}+\beta_{7}Marstat_{\mathrm{it}}\\ +\beta_{8}Child_{\mathrm{it}}+\beta_{9}Part_{\mathrm{it}}+\mu_{i}+\varepsilon_{\mathrm{it}} \end{array}$$

The model includes the five 24‐months splines, dummy variables for the class of origin, ethnic origin, fields of study, type of degree, marital status, part‐time employment and linear specifications for cognitive ability and the number of children. It also contains a person‐specific unobserved factor μ (random effect) and a time‐varying error term ε. The intraclass correlation – which we calculate based on the variances of the error terms $\left(\rho =\sigma_{\mu }^{2}/\left(\sigma_{\mu }^{2}+\sigma_{\varepsilon }^{2}\right)\right)$ – shows us how much of the overall variance in occupational prestige is due to variation between individuals and due to variation within individuals across the early and mid‐career. The stronger this intraclass correlation, the more inequality between graduates in occupational prestige exists, and the fewer within‐changes we can identify across graduate careers.

The $\beta_{1s}$ estimates indicate the average monthly change in occupational attainment within the 24‐month splines. The remaining β coefficients indicate the strength of associations between the respective variables and occupational prestige averaged across the early working career. To test our first hypothesis, we extend our baseline model by including interaction terms between graduates’ class of origin and our five splines for time since labour market entry. To test the hypotheses on field‐specific social inequalities, we run our extended model, including the interaction between graduates’ class of origin and spline for the three different field groups separately.

## Results

### Occupational prestige across the early career

Table 2 indicates a series of growth curve models. To decompose the total variance into variance between individuals and within‐variance across the career, our first model (M0) is an empty model without any covariates. Model M1 introduces five 24‐months splines to show how occupational prestige changes over the first ten years after labour market entry. Model M2 further includes the class of origin, ethnicity, gender, cognitive ability, and the graduate characteristics (field of study, type of degree). It also accounts for the family variables and an indicator of part‐time employment.

**Table 2:** Summary of growth curve models predicting occupational prestige (*N* = 99,993 person‐months from 951 graduates)

	M0	M1	M2
24 months (2 yrs) or less		0.126***	0.120***
		(0.015)	(0.015)
25–48 months (3–4 yrs)		0.043***	0.045***
		(0.012)	(0.013)
49–72 months (5–6 yrs)		0.027*	0.030**
		(0.010)	(0.011)
73–96 months (7–8 yrs)		0.017	0.024*
		(0.011)	(0.012)
97–120 months (9–10 yrs)		0.021*	0.027*
		(0.010)	(0.011)
Female (ref. male)			‐0.291
			(0.822)
Non‐UK origin (ref. UK)			0.586
			(1.652)
Parental class (ref. Salariat class)			
Intermediate class			0.018
			(0.679)
Working class			‐0.655
			(0.945)
Cognitive ability			0.120***
			(0.027)
Field of study (ref. humanities)			
Social sciences			‐0.697
			(2.619)
STEM fields			‐0.717
			(2.184)
Law and medicine			5.598
			(2.963)
Postgraduate (ref. undergraduate)			4.635*
			(1.976)
Intercept	54.010***	50.160***	50.445***
	(0.314)	(0.464)	(1.951)
*Variance components*			
Between‐individual	93.257	91.980	82.235
Within‐individual	38.267	36.236	35.666
Intraclass correlation (rho)	0.71	0.72	0.70
Chi^2^		133.36	215.61

Note:

Model M2 additionally controls for marital status, number of children and part‐time employment (estimates not shown). Robust standard errors are in parentheses.

* *p* < 0.05,

** *p* < 0.01,

*** *p* < 0.001.

The intraclass correlation coefficient (ICT) as shown in M0 indicates to what extent the total variation in occupational prestige is due to differences between graduates rather than within‐differences across the career. In our sample, the ICT shows that 71 per cent of the overall variance is due to differences in occupational prestige between graduates. Thus, differences in occupational prestige between graduates instead emerge at the beginning of the career, and there is only modest career mobility over time that corrects for these initial differences. Compared to working populations in Germany (Manzoni et al. 2014) or Italy (Barone, Lucchini and Schizzerotto 2011), however, the ICT is smaller, that is, career mobility (among graduates) is higher than in these settings. It may indicate that the UK labour market is more dynamic than labour markets in other countries or that graduate labour markets offer more leeway for career progression or both.

Model 1 in Table 2 introduces the five 24‐month splines. Graduates make progress in their career predominantly in the first two years after they gained their first significant job: The average graduate gains 3.02 (24 × 0.126) SIOPS points across the first 24 months. In the following two years, career progression slows down but graduates, on average, still achieve considerable gains (1.032 SIOPS points, 24 × 0.043). After four years since the first significant job, graduates appear to be more established in their careers and change in occupational prestige is limited but not entirely stopped. Graduates, on average, gain 1.56 SIOPS points (24 × 0.027 + 24 × 0.017 + 24 × 0.021) across the following six years. Overall, graduates gain, on average, 5.6 SIOPS points across the first ten years since labour market entry.

Model 2 includes our main variable of interest: the class of origin. Holding confounders constant, graduates from an intermediate class background gain, on average, 0.018 SIOPS points and working‐class background gain, on average, 0.655 SIOPS points less than graduates whose parents belong to the salariat class. Hence, averaged across graduate careers, the direct effect of the class of origin on occupational prestige net of cognitive ability and horizontal educational differences appears to be rather small.

### Occupational career progression by social origin and field of study

**Figure 2:**
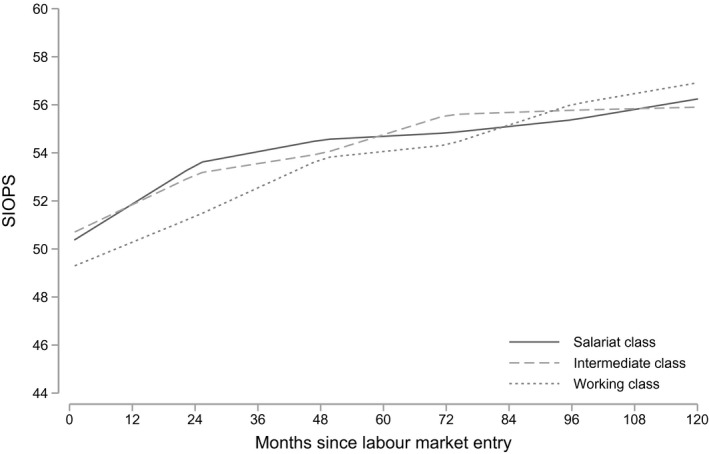
Predicted occupational prestige by parental class

**Figure 3:**
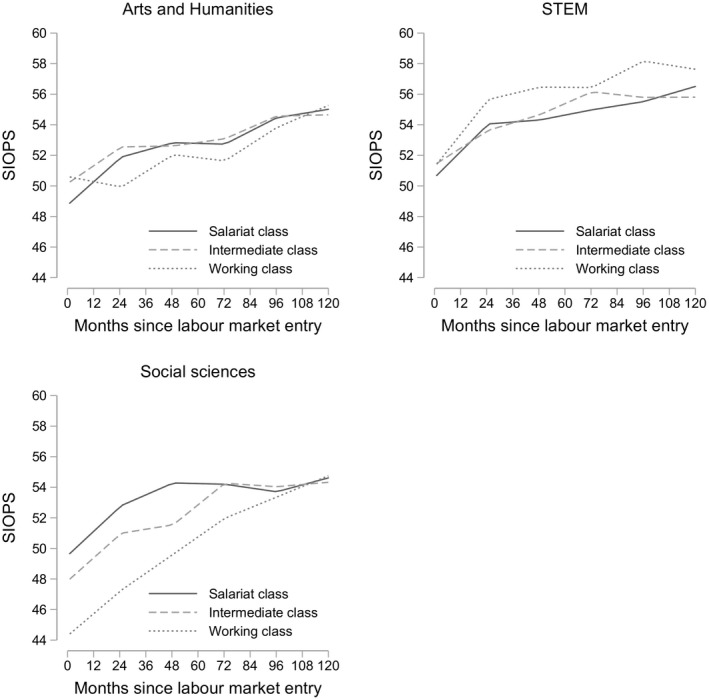
Predicted occupational prestige by parental class across different fields of study

So far, the effects of career progression analysed in Table 2 refer to average careers. We now turn to the results on career progression by the class of origin as expressed in hypothesis 1. Figure 2 shows the predicted SIOPS scores across the career for graduates from different classes of origin (see full estimates in Appendix Table A.II). In this figure, we can see small differences in occupational prestige between graduates from working‐class background and graduates from both intermediate and salariat class background at labour market entry. This difference becomes slightly stronger across the first two years as graduates from salariat and intermediate class backgrounds have steeper growth curves than graduates from a working‐class background. At two years after labour market entry, graduates from the service class have an advantage in occupational prestige of around one prestige point on the SIOPS scale over graduates from the working class.^4^ A one‐point difference in occupational prestige is, for instance, equivalent to the difference between a biologist (SIOPS score 69) and a pharmacologist (SIOPS score 68), or the difference between a writer (SIOPS score 58) and a painter (SIOPS score 57) (Figure 3). Over the following two years, occupational prestige continues to grow to a similar extent for working‐class graduates, while growth slows down for graduates from higher‐class backgrounds. Hence, graduates from working‐class backgrounds catch up with their peers from more advantaged backgrounds after four years in the labour market.

To test our field‐specific hypothesis, we predicted SIOPS scores by the class of origin and time since labour market entry for the different field of study groups separately (see full estimates in Appendix Table A.III), results are shown in Figure 3. In humanities, the class of origin differences are minimal. If at all, working‐class graduates tend to be disadvantaged in their career progression in the first two years after gaining a first significant job but they catch up with their peers from more advantaged backgrounds later on. Likewise, we do not find substantial social inequalities in career progression for graduates from STEM fields. Working‐class graduates achieve slightly higher scores of occupational prestige at all stages of their career. In contrast, working‐class graduates in the social sciences have, on average, a prestige score at labour market entry that is about five SIOPS points lower than the prestige score of graduates from more advantaged backgrounds. Graduates from working‐class backgrounds can overcome this initial disadvantage and catch up with their peers from higher‐class backgrounds eight years after graduation.

We replicated our analysis with a different outcome using the International Socio‐Economic Index of occupational status (ISEI) (see Appendix Figures A.I and A.II). The results largely confirm our previous analysis. As with occupational prestige, we only find disadvantages in socio‐economic status for graduates from a working‐class background in the social sciences. As in our main analysis, these inequalities in status vanish across the early labour market career. Our results also hold when including part‐time students in our graduate sample (see Appendix Figures A.II and A.III). Other than in our primary analysis, however, social inequalities among graduates from humanities emerge after the initial labour market entry but decrease again at the end of our observation period.

## Discussion

We examined early occupational careers of degree‐holders in the UK and the long‐term influence of social origin up to 10 years after labour market entry. Differentiating between graduates of different fields allowed us to describe the evolution of social inequalities on top of the educational level. Given the increasing share of degree‐holders in the UK (and elsewhere) qualitative differences such as field of study may have become increasingly crucial for processes of social stratification. By adopting a dynamic approach looking at intergenerational occupational mobility, we wanted to detect whether and to what extent social inequalities in the early career are strengthened or mitigated, and whether initial disparities and differences in career progression vary by field of study.

We did not find a substantial direct effect of social origin on graduates’ average occupational attainment throughout ten years after labour market entry. This finding is in line with previous results on UK graduates using the same dataset (e.g., most recently, Sullivan et al. 2018a, 2018b). However, when we looked more closely at the effect of social origin on occupational prestige at different stages of the career, that is, extending previous snapshot analyses with a dynamic approach, our illustration of career patterns showed a slight disadvantage of working‐class offspring compared to graduates from the higher social origin at labour market entry. Due to a steeper progression of graduates from a working‐class background, however, this disadvantage vanishes across the early career, and they achieve about the same level of occupational prestige as their peers from advantaged backgrounds.

This result provides evidence for our first hypothesis (see also Bukodi and Goldthorpe 2011; Jacob, Klein and Iannelli 2015). In line with our theoretical considerations, this suggests that graduates from working‐class backgrounds can correct for initial occupational mismatches as graduates’ work experience becomes more relevant in occupational allocation at later stages of the career. Regarding the development of occupational prestige in different fields of study, we saw that social class differences in career growth vary by field of study. In line with our expectations, we found social inequalities in occupational prestige neither at labour market entry nor in career progression in STEM fields. As STEM fields provide students with occupation‐specific qualifications, there seems to be less leeway for the parental background to interfere.

Contrary to our assumptions, there were also no significant social gradients in occupational careers for graduates in arts and humanities. This implies that the larger endowment with cultural and social resources among graduates from higher socio‐economic backgrounds makes no difference for occupational attainment neither at labour market entry nor for career progression. At this point, we can only speculate about alternative explanations. Graduates from humanities may enter specific labour market segments with low variation in occupational prestige so that potential advantages of service class children cannot be realized. It may also be the case that working‐class graduates in humanities are a particularly selective group, that is, they are quite similar to those from service class backgrounds regarding unobserved characteristics.

We observed pronounced inequalities among graduates in the social sciences but in the opposite way that we expected. Working‐class graduates attain significantly lower occupational prestige at labour market entry compared to graduates from higher social origins. However, they catch up with their peers and achieve the same level of occupational prestige a few years after the labour market entry. Hence, the results indicate that the mechanisms we expected for graduates from arts and humanities may instead apply to graduates in the social sciences. Graduates from the social sciences may have a high risk of being over‐educated at labour market entry, and parental resources protect graduates from advantaged backgrounds from having to accept a vertically mismatched position. Parental social and cultural capital may, therefore, be essential to gain access to prestigious entry positions for social science graduates. Over time, parental resources may play less of a role in job allocation procedures as graduates’ real productivity becomes visible. The catching‐up process indicates that factors (e.g., social and soft skills) that are advantageous for initial occupational positions of graduates from higher classes do not provide a persistent advantage over graduates from working‐class origin throughout the career. It remains open for future research with richer data to test the mechanisms empirically behind the career patterns we have observed. Summing up, our results support the claim that an undergraduate degree is an ‘equalizer’ for labour market outcomes as social origin has no effect on occupational prestige over and above attaining a degree in the long run (Torche 2011). Whereas working class children that achieve a degree may be a selective group of graduates with productivity‐enhancing characteristics, recent research suggests that the weak association between social origin and labour market outcomes is not due to their selectivity (Karlson 2019). Due to a small number of cases, we were not able to test whether we see a U‐shaped pattern of parental influence and social inequalities emerge again more strongly when considering individuals with postgraduate degrees. Our results also highlight the importance of considering heterogeneity in social inequalities in the labour market within broader educational groups. Field‐specific variation in the social gradient in occupational prestige is hidden when looking at the average association between social origin and graduate careers.

From a broader theoretical point of view, our results provide evidence for signalling theory rather than human capital theory. If graduates from higher social origin gained skills through their upbringing such as soft or social skills that increase their productivity in the labour market, they should have advantages both at labour market entry and in later career stages. However, our results show that social inequalities among graduates from the social sciences are limited to the entry stage. It suggests if at all that employers in managerial, sales and personal service occupations use skills that are associated to the social origin as a signal for future productivity when more reliable indicators of work performance are missing.

Our paper has several limitations: First, our study is merely descriptive in analysing the career trajectories of graduates from different social backgrounds. A causal interpretation of our estimates rests on the strong assumption that there are no important unmeasured factors that influence degree attainment in different fields of study, occupational attainment, and its progression, and at the same time vary by social origin. Other than previous studies focusing on graduates, our study, however, accounts for cognitive ability differences between graduates from different backgrounds and as such may address a considerable part of the selectivity in our graduate sample.

Our second limitation is the weak statistical power primarily when analysing social inequalities in career progression across fields of study. Compared to existing graduate surveys, our birth cohort panel data provide the advantage of looking at graduates’ long‐term career mobility in a more dynamic perspective but at the cost of having smaller  sample sizes. Therefore, we need to consider our results with caution, and future research needs to replicate these results with larger graduate samples.

Third, our paper focused on the outcome of occupational prestige and as such, only detected between‐occupational changes across the working career. However, graduates also improve their labour market outcomes by changing positions within occupations. Extending our analysis with income seems to be a promising endeavour for future research as it takes into account within‐occupational career changes and is less prone to ceiling effects. Hence, we cannot exclude the possibility that inter‐ and intra‐generational mobility patterns may be different from what we observe in our analysis when using income (Sullivan et al. 2018b; Witteveen and Attewell 2017 for the US).

Finally, we did not assess career progression beyond ten years after labour market entry. We cannot rule out the notion that social inequalities emerge again in the later stages of the life course. Also, social disparities may go beyond occupational stratification, that is, emerge when considering different labour market sectors, different employment statuses, and different geographic locations as has been examined by Manzoni and Streib (2018) in the US context.

Given the insights gained by applying a dynamic perspective on the career progression of graduates, we suggest that longitudinal approaches on intergenerational mobility need to be further developed, both theoretically and empirically, to gain a better understanding of underlying mechanisms on when and how social inequalities emerge, increase or even decline over the life‐course. It is also crucial to develop a more thorough theoretical approach as to where and to what extent social class differences in labour market destinations may arise among different educational or labour market subgroups.

## Supporting information


**Part I**
**Table A.I:** Aggregation of fields of study into three main groups
**Table A.II:** Growth curve models predicting occupational prestige by class of origin (N = 99,993 person‐months from 951 graduates)
**Table A.III:** Growth curve models predicting occupational prestige by class of origin for humanities (N = 29,355 person‐months from 289 graduates), social sciences (N = 31,572 person‐months from 308 graduates) and STEM (N = 32,287 person‐months from 312 graduates)
**Part II:** ISEI
**Table A.IV:** Growth curve models predicting ISEI by class of origin (N = 100,233 person‐months from 955 graduates)
**Table A.V:** Growth curve models predicting ISEI by class of origin for humanities (N = 29,601 person‐months from 290 graduates), social sciences (N = 31,878 person‐months from 310 graduates) and STEM (N = 32,569 person‐months from 313 graduates)
**Figure A.I:** Predicted ISEI by parental class
**Figure A.II:** Predicted ISEI by parental class across different fields of study
**Part III:** Including part‐time students
**Table A.VI:** Growth curve models predicting ISEI by class of origin (N = 124,148 person‐months from 1209 graduates)
**Table A.VII:** Growth curve models predicting ISEI by class of origin for humanities (N = 32,181 person‐months from 320 graduates), social sciences (N = 45,042 person‐months from 456 graduates) and STEM (N = 38,931 person‐months from 385 graduates)
**Figure A.III:** Predicted SIOPS by parental class
**Figure A.IV: **Predicted SIOPS by parental class across different fields of study

## Notes

2 The analyses of Lambert and Bihagen (2014) show that occupational measures like ISEI (combination of occupational income and typical educational level (Ganzeboom and Treiman 1996), occupational prestige (Treiman 1977) and earnings growth (Bihagen and Ohls 2007) are highly correlated (see also Bihagen and Lambert 2018 for class and social status). Besides, Warren et al. (1998) point out further advantages of prestige scores when analysing labour market outcomes of women. Finally, in survey data, occupational prestige is a more reliable measure than income and is less affected by item non‐response.

3 We consider law and medicine as a separate category because their professional pathways are more standardized and do not involve many between‐occupation changes. Unfortunately, we cannot consider this category in our field‐specific analysis of social inequalities in career progression as the number of cases is too small to conduct such an analysis.

4 To put this in perspective, West German men from the upper service class are, on average, seven prestige points ahead of men from working‐class backgrounds once adjusting for educational attainment (Manzoni et al. 2014). This direct effect of social origin on occupational prestige is presumably larger in the UK as previous studies suggest a stronger direct effect in the UK than in other European countries (Breen and Luijkx 2004).
