# Supplementary material for: Social origin, field of study and graduates’ career progression: does social inequality vary across fields?
Source: Br J Sociol. 2019 Aug 14;70(5):1850–73. doi: 10.1111/1468-4446.12696 (PMC6916604; doi:10.1111/1468-4446.12696)
Supplement: Supplementary file 1 — Part I Table A.I: Aggregation of fields of study into three main groups Table A.II: Growth curve models predicting occupational prestige by class of origin (N = 99,993 person‐months from 951 graduates) Table A.III: Growth curve models predicting occupational prestige by class of origin for humanities (N = 29,355 person‐months from 289 graduates), social sciences (N = 31,572 person‐months from 308 graduates) and STEM (N = 32,287 person‐months from 312 graduates) Part II: ISEI Table A.IV: Growth curve models predicting ISEI by class of origin (N = 100,233 person‐months from 955 graduates) Table A.V: Growth curve models predicting ISEI by class of origin for humanities (N = 29,601 person‐months from 290 graduates), social sciences (N = 31,878 person‐months from 310 graduates) and STEM (N = 32,569 person‐months from 313 graduates) Figure A.I: Predicted ISEI by parental class Figure A.II: Predicted ISEI by parental class across different fields of study Part III: Including part‐time students Table A.VI: Growth curve models predicting ISEI by class of origin (N = 124,148 person‐months from 1209 graduates) Table A.VII: Growth curve models predicting ISEI by class of origin for humanities (N = 32,181 person‐months from 320 graduates), social sciences (N = 45,042 person‐months from 456 graduates) and STEM (N = 38,931 person‐months from 385 graduates) Figure A.III: Predicted SIOPS by parental class Figure A.IV: Predicted SIOPS by parental class across different fields of study [file BJOS-70-1850-s001.docx]

**Online Appendix**

**PART I**

Table AI. *Aggregation of fields of study into three main groups*

| *Humanities* | *Social Sciences* | *STEM* | *Law and medicine* |
| --- | --- | --- | --- |
| English | Accountancy | Architecture | Law |
| Art | Psychology | Engineering | Medicine |
| Linguistics | Statistics | Agriculture | Dentistry |
| American studies | Applied social sciences | Science |  |
| Ancient History | Business | Biology |  |
| Applied Language Studies | Economics | Chemistry |  |
| Drama | Banking, insurance and finance | Geology |  |
| Film, radio and television | Behavioural science | Physics |  |
| Textile Design | Estate management | Mechanical Engineering |  |
| Acting studies | Geography | Computer Science |  |
| Archaeology | Health studies | Biochemistry |  |
| Arts & Design | Management | Math |  |
| Education | Pharmacology | Astronomy |  |
| Graphic design | Physiology | Building Engineering |  |
| Community studies | Physiotherapy | Civil Engineering |  |
| Cultural studies | Political science | Construction |  |
| Classical civilisation | Public policy | Electronics |  |
| Communication studies | Sociology | Environmental Science |  |
| Dance studies | Social policy | Information Technology |  |
| Design illustration | Social anthropology | Manufacturing Engineering |  |
| European languages | Urban studies | Marine Biology |  |
| Fashion design | Zoology | Mechanical Engineering |  |
| Fine art | Anatomy | Cell and Molecular Biology |  |
| French | Animal science | Earth sciences |  |
| German | Marketing | Ecology |  |
| Hispanic studies | Nursing studies | Technology |  |
| History | Pathology | Telecommunications |  |
| Libriarianship | Public administration |  |  |
| Media studies | Social work |  |  |
| Modern studies |  |  |  |
| Music |  |  |  |
| Performing arts |  |  |  |
| Philosophy |  |  |  |
| Physical education |  |  |  |
| Religion |  |  |  |
| Spanish |  |  |  |
| Sport studies |  |  |  |
| Theology |  |  |  |
| Classical music |  |  |  |
| Photography |  |  |  |

Table AII. *Growth curve models predicting occupational prestige by class of origin (N = 99,993 person-months from 951 graduates)*

|  | **M3** |
| --- | --- |
| 24 months (2 yrs) or less | 0.135^***^ |
|  | (0.022) |
| 25-48 months (3-4 yrs) | 0.040^**^ |
|  | (0.016) |
| 49-72 months (5-6 yrs) | 0.012 |
|  | (0.014) |
| 73-96 months (7-8 yrs) | 0.023 |
|  | (0.017) |
| 97-120 months (9-10 yrs) | 0.036^*^ |
|  | (0.014) |
| Female (ref. male) | -0.284 |
|  | (0.832) |
| Non-UK origin (ref. UK) | 0.643 |
|  | (1.664) |
| Parental class (ref. Salariat class) |  |
| Intermediate class | 0.356 |
|  | (0.999) |
| Working class | -1.039 |
|  | (1.420) |
| Cognitive ability | 0.121^***^ |
|  | (0.027) |
| Field of study (ref. humanities) |  |
| Social sciences | -0.731 |
|  | (2.655) |
| STEM fields | -0.672 |
|  | (2.708) |
| Law and medicine | 5.082 |
|  | (3.027) |
| Postgraduate (ref. undergraduate) | 4.703^*^ |
|  | (1.960) |
| Intermed. class x 24months or less | -0.032 |
|  | (0.032) |
| Intermed. class * 25-48months | -0.006 |
|  | (0.025) |
| Intermed. class * 49-72months | 0.056^*^ |
|  | (0.023) |
| Intermed. class * 73-96months | -0.006 |
|  | (0.025) |
| Intermed. class * 97-120months | -0.015 |
|  | (0.024) |
| Working class * 24months | -0.046 |
|  | (0.044) |
| Working class * 25-48months | 0.059 |
|  | (0.043) |
| Working class * 49-72months | 0.011 |
|  | (0.034) |
| Working class * 73-96months | 0.048 |
|  | (0.035) |
| Working class * 97-120months | 0.001 |
|  | (0.036) |
| Intercept | 50.419^***^ |
|  | (2.040) |
| *Variance components* |  |
| Between-individual | 82.636 |
| Within-individual | 35.560 |
| Intraclass correlation (rho) | 0.70 |
| Chi^2^ | 223.91 |

*Source:* British Cohort Study 1970 (BCS70); *Note:* model controls for marital status, number of children and part-time employment. Robust standard errors are in parentheses. ^*^ *p* < 0.05, ^**^ *p* < 0.01, ^***^ *p* < 0.001.

Table AIII. *Growth curve models predicting occupational prestige by class of origin for humanities (N=29,355 person-months from 289 graduates), social sciences (N=31,572 person-months from 308 graduates) and STEM (N=32,287 person-months from 312 graduates)*

|  | **M4 (Humanities)** | **M5 (Social sciences)** | **M6 (STEM)** |
| --- | --- | --- | --- |
| 24 months (2 yrs) or less | 0.127^**^ | 0.132^***^ | 0.142^***^ |
|  | (0.040) | (0.039) | (0.039) |
| 25-48 months (3-4 yrs) | 0.039 | 0.062^*^ | 0.011 |
|  | (0.029) | (0.030) | (0.026) |
| 49-72 months (5-6 yrs) | -0.004 | -0.003 | 0.028 |
|  | (0.026) | (0.023) | (0.025) |
| 73-96 months (7-8 yrs) | 0.072^**^ | -0.021 | 0.023 |
|  | (0.026) | (0.033) | (0.030) |
| 97-120 months (9-10 yrs) | 0.023 | 0.038 | 0.042 |
|  | (0.024) | (0.029) | (0.023) |
| Female (ref. male) | 0.838 | 0.195 | -0.707 |
|  | (1.271) | (1.094) | (1.269) |
| Non-UK origin (ref. UK) | -0.705 | 2.365 | -1.514 |
|  | (2.727) | (3.301) | (2.159) |
| Parental class (ref. Salariat class) |  |  |  |
| Intermediate class | 1.409 | -1.664 | 0.836 |
|  | (1.699) | (1.827) | (1.756) |
| Working class | 1.871 | -5.247^*^ | 0.695 |
|  | (2.527) | (2.128) | (2.817) |
| Cognitive ability | 0.119^*^ | 0.083 | 0.120^**^ |
|  | (0.047) | (0.051) | (0.044) |
| Postgraduate (ref. undergraduate) | -7.608^***^ | 3.361 | 6.875 |
|  | (1.326) | (2.632) | (3.930) |
| Intermed. class * 24m | -0.030 | -0.006 | -0.051 |
|  | (0.063) | (0.057) | (0.054) |
| Intermed. class * 25-48m | -0.037 | -0.039 | 0.032 |
|  | (0.040) | (0.046) | (0.042) |
| Intermed. class * 49-72m | 0.024 | 0.118^**^ | 0.035 |
|  | (0.038) | (0.040) | (0.042) |
| Intermed. class * 73-96m | -0.010 | 0.011 | -0.038 |
|  | (0.038) | (0.052) | (0.040) |
| Intermed. class * 97-120m | -0.020 | -0.026 | -0.041 |
|  | (0.044) | (0.045) | (0.032) |
| Working class * 24m | -0.155^**^ | -0.012 | 0.037 |
|  | (0.053) | (0.080) | (0.090) |
| Working class * 25-48m | 0.050 | 0.037 | 0.023 |
|  | (0.082) | (0.065) | (0.056) |
| Working class * 49-72m | -0.013 | 0.103 | -0.029 |
|  | (0.062) | (0.069) | (0.043) |
| Working class * 73-96m | 0.019 | 0.079 | 0.050 |
|  | (0.067) | (0.064) | (0.056) |
| Working class * 97-120m | 0.038 | 0.020 | -0.064 |
|  | (0.058) | (0.051) | (0.073) |
| Intercept | 49.212^***^ | 49.378^***^ | 49.366^***^ |
|  | (1.501) | (1.241) | (1.575) |
| *Variance components* |  |  |  |
| Between-individual | 79.422 | 80.114 | 80.777 |
| Within-individual | 32.187 | 39.207 | 32.344 |
| Intraclass correlation (rho) | 0.71 | 0.67 | 0.71 |
| Chi^2^ | 80.99 | 100.59 | 90.94 |

*Source:* British Cohort Study 1970 (BCS70); *Note:* All models control for marital status, number of children and part-time employment. Robust standard errors are in parentheses. ^*^ *p* < 0.05, ^**^ *p* < 0.01, ^***^ *p* < 0.001.

**PART II: ISEI**

Table AIV. *Growth curve models predicting ISEI by class of origin (N = 100,233 person-months from 955 graduates)*

|  | **M3** |
| --- | --- |
| 24 months (2 yrs) or less | 0.129^***^ |
|  | (0.025) |
| 25-48 months (3-4 yrs) | 0.073^***^ |
|  | (0.019) |
| 49-72 months (5-6 yrs) | 0.019 |
|  | (0.016) |
| 73-96 months (7-8 yrs) | 0.022 |
|  | (0.019) |
| 97-120 months (9-10 yrs) | 0.043^*^ |
|  | (0.017) |
| Female (ref. male) | -1.768 |
|  | (1.141) |
| Non-UK origin (ref. UK) | 1.461 |
|  | (2.112) |
| Parental class (ref. Salariat class) |  |
| Intermediate class | 0.284 |
|  | (1.186) |
| Working class | 0.350 |
|  | (1.595) |
| Cognitive ability | 0.141^***^ |
|  | (0.034) |
| Field of study (ref. humanities) |  |
| Social sciences | 0.886 |
|  | (3.225) |
| STEM fields | -1.062 |
|  | (3.136) |
| Law and medicine | 9.072^**^ |
|  | (3.454) |
| Postgraduate (ref. undergraduate) | 6.492^**^ |
|  | (2.392) |
| Intermed. class * 24months or less | 0.003 |
|  | (0.037) |
| Intermed. class * 25-48months | -0.029 |
|  | (0.029) |
| Intermed. class * 49-72months | 0.058^*^ |
|  | (0.027) |
| Intermed. class * 73-96months | -0.024 |
|  | (0.028) |
| Intermed. class * 97-120months | -0.032 |
|  | (0.027) |
| Working class * 24months | -0.044 |
|  | (0.045) |
| Working class * 25-48months | -0.009 |
|  | (0.048) |
| Working class * 49-72months | 0.019 |
|  | (0.041) |
| Working class * 73-96months | 0.057 |
|  | (0.047) |
| Working class * 97-120months | -0.006 |
|  | (0.040) |
| Intercept | 56.068^***^ |
|  | (2.040) |
| *Variance components* |  |
| Between-individual | 129.644 |
| Within-individual | 48.938 |
| Intraclass correlation (rho) | 0.73 |
| Chi^2^ | 236.86 |

*Source:* British Cohort Study 1970 (BCS70); *Note:* model controls for marital status, number of children and part-time employment. Robust standard errors are in parentheses. ^*^ *p* < 0.05, ^**^ *p* < 0.01, ^***^ *p* < 0.001.

Table AV. *Growth curve models predicting ISEI by class of origin for humanities (N=29,601 person-months from 290 graduates), social sciences (N=31,878 person-months from 310 graduates) and STEM (N=32,569 person-months from 313 graduates)*

|  | **M4 (Humanities)** | **M5 (Social sciences)** | **M6 (STEM)** |
| --- | --- | --- | --- |
| 24 months (2 yrs) or less | 0.122^**^ | 0.141^***^ | 0.116^*^ |
|  | (0.045) | (0.046) | (0.039) |
| 25-48 months (3-4 yrs) | 0.071 | 0.091^**^ | 0.043 |
|  | (0.039) | (0.034) | (0.027) |
| 49-72 months (5-6 yrs) | -0.001 | -0.010 | 0.051 |
|  | (0.031) | (0.027) | (0.027) |
| 73-96 months (7-8 yrs) | 0.060^*^ | -0.021 | 0.021 |
|  | (0.028) | (0.032) | (0.040) |
| 97-120 months (9-10 yrs) | 0.028 | 0.046 | 0.044 |
|  | (0.025) | (0.036) | (0.031) |
| Female (ref. male) | 1.484 | -1.662 | -2.621 |
|  | (1.619) | (1.291) | (1.590) |
| Non-UK origin (ref. UK) | -3.116 | 5.456 | -0.935 |
|  | (4.221) | (3.285) | (3.006) |
| Parental class (ref. Salariat class) |  |  |  |
| Intermediate class | 1.447 | -2.663 | 0.631 |
|  | (2.021) | (2.041) | (2.139) |
| Working class | 1.657 | -3.546 | 3.085 |
|  | (2.706) | (2.585) | (2.981) |
| Cognitive ability | 0.123^*^ | 0.110 | 0.121^*^ |
|  | (0.058) | (0.051) | (0.058) |
| Postgraduate (ref. undergraduate) | -12.115^***^ | 1.641 | 11.600^*^ |
|  | (1.514) | (2.677) | (4.579) |
| Intermed. class * 24m | -0.010 | -0.004 | 0.034 |
|  | (0.070) | (0.053) | (0.050) |
| Intermed. class * 25-48m | -0.070 | -0.044 | 0.020 |
|  | (0.050) | (0.053) | (0.047) |
| Intermed. class * 49-72m | 0.019 | 0.139^**^ | 0.035 |
|  | (0.043) | (0.052) | (0.042) |
| Intermed. class * 73-96m | 0.012 | 0.008 | -0.055 |
|  | (0.039) | (0.056) | (0.053) |
| Intermed. class * 97-120m | -0.035 | -0.019 | -0.031 |
|  | (0.044) | (0.056) | (0.044) |
| Working class * 24m | -0.125^*^ | -0.078 | 0.077 |
|  | (0.054) | (0.082) | (0.090) |
| Working class * 25-48m | -0.049 | -0.001 | -0.046 |
|  | (0.091) | (0.073) | (0.053) |
| Working class * 49-72m | 0.040 | 0.126 | -0.078 |
|  | (0.078) | (0.078) | (0.047) |
| Working class * 73-96m | 0.033 | 0.127 | 0.036 |
|  | (0.089) | (0.091) | (0.070) |
| Working class * 97-120m | 0.027 | -0.017 | -0.064 |
|  | (0.065) | (0.074) | (0.054) |
| Intercept | 54.361^***^ | 55.124^***^ | 55.687^***^ |
|  | (1.841) | (1.435) | (1.829) |
| *Variance components* |  |  |  |
| Between-individual | 128.707 | 114.466 | 129.433 |
| Within-individual | 42.974 | 50.664 | 47.840 |
| Intraclass correlation (rho) | 0.75 | 0.69 | 0.73 |
| Chi^2^ | 101.62 | 99.70 | 95.04 |

*Source:* British Cohort Study 1970 (BCS70); *Note:* All models control for marital status, number of children and part-time employment. Robust standard errors are in parentheses. ^*^ *p* < 0.05, ^**^ *p* < 0.01, ^***^ *p* < 0.001.

FIGURE AI. PREDICTED ISEI BY PARENTAL CLASS


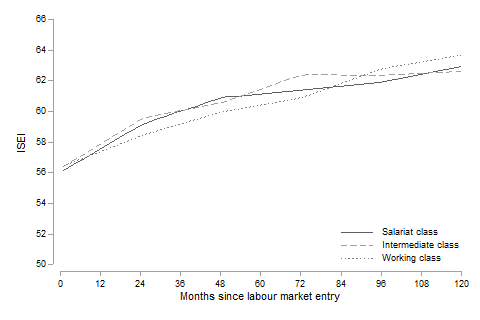


*Source:* British Cohort Study 1970 (BCS70); *Note:* Estimates obtained from growth curve model 5 in Appendix Table AIV.

FIGURE AII. PREDICTED ISEI BY PARENTAL CLASS ACROSS DIFFERENT FIELDS OF STUDY

*Source:* British Cohort Study 1970 (BCS70); *Note:* Respective estimates obtained from models 6-8 in Online Appendix Table AV.

**PART III: Including part-time students**

Table AVI. *Growth curve models predicting ISEI by class of origin (N = 124,148 person-months from 1209 graduates)*

|  | **M3** |
| --- | --- |
| 24 months (2 yrs) or less | 0.127^***^ |
|  | (0.019) |
| 25-48 months (3-4 yrs) | 0.037^**^ |
|  | (0.014) |
| 49-72 months (5-6 yrs) | 0.009 |
|  | (0.013) |
| 73-96 months (7-8 yrs) | 0.023 |
|  | (0.017) |
| 97-120 months (9-10 yrs) | 0.047^**^ |
|  | (0.014) |
| Female (ref. male) | -0.113 |
|  | (0.712) |
| Non-UK origin (ref. UK) | 0.117 |
|  | (1.424) |
| Parental class (ref. Salariat class) |  |
| Intermediate class | 0.482 |
|  | (0.851) |
| Working class | -1.341 |
|  | (1.206) |
| Cognitive ability | 0.096^***^ |
|  | (0.026) |
| Field of study (ref. humanities) |  |
| Social sciences | -0.521 |
|  | (2.773) |
| STEM fields | 0.437 |
|  | (2.268) |
| Law and medicine | 11.452^***^ |
|  | (3.069) |
| Postgraduate (ref. undergraduate) | 3.700^*^ |
|  | (1.560) |
| Part-time studies (ref. full-time studies) | 0.790 |
|  | (2.004) |
| Intermed. class * 24months or less | -0.036 |
|  | (0.029) |
| Intermed. class * 25-48months | -0.008 |
|  | (0.022) |
| Intermed. class * 49-72months | 0.045^*^ |
|  | (0.021) |
| Intermed. class * 73-96months | -0.011 |
|  | (0.023) |
| Intermed. class * 97-120months | -0.032 |
|  | (0.022) |
| Working class * 24months | -0.063 |
|  | (0.037) |
| Working class * 25-48months | 0.034 |
|  | (0.036) |
| Working class * 49-72months | 0.015 |
|  | (0.029) |
| Working class * 73-96months | 0.037 |
|  | (0.033) |
| Working class * 97-120months | -0.009 |
|  | (0.036) |
| Intercept | 50.319^***^ |
|  | (1.986) |
| *Variance components* |  |
| Between-individual | 76.965 |
| Within-individual | 35.252 |
| Intraclass correlation (rho) | 0.69 |
| Chi^2^ | 274.00 |

*Source:* British Cohort Study 1970 (BCS70); *Note:* model controls for marital status, number of children and part-time employment. Robust standard errors are in parentheses. ^*^ *p* < 0.05, ^**^ *p* < 0.01, ^***^ *p* < 0.001.

Table AVII. *Growth curve models predicting ISEI by class of origin for humanities (N=32,181 person-months from 320 graduates), social sciences (N=45,042 person-months from 456 graduates) and STEM (N=38,931 person-months from 385 graduates)*

|  | **M4 (Humanities)** | **M5 (Social sciences)** | **M6 (STEM)** |
| --- | --- | --- | --- |
| 24 months (2 yrs) or less | 0.127^**^ | 0.125^***^ | 0.125^***^ |
|  | (0.038) | (0.031) | (0.034) |
| 25-48 months (3-4 yrs) | 0.044 | 0.049^*^ | 0.014 |
|  | (0.028) | (0.024) | (0.023) |
| 49-72 months (5-6 yrs) | -0.002 | -0.002 | 0.051 |
|  | (0.026) | (0.019) | (0.027) |
| 73-96 months (7-8 yrs) | 0.066^*^ | -0.009 | 0.021 |
|  | (0.029) | (0.031) | (0.024) |
| 97-120 months (9-10 yrs) | 0.028 | 0.059^*^ | 0.045 |
|  | (0.025) | (0.028) | (0.025) |
| Female (ref. male) | 0.528 | -0.268 | -0.179 |
|  | (1.189) | (0.876) | (1.139) |
| Non-UK origin (ref. UK) | -0.204 | 1.010 | -1.427 |
|  | (2.467) | (2.236) | (2.275) |
| Parental class (ref. Salariat class) |  |  |  |
| Intermediate class | 1.112 | -0.470 | 1.225 |
|  | (1.616) | (1.358) | (1.575) |
| Working class | 0.429 | -3.073 | 0.233 |
|  | (2.416) | (1.669) | (2.441) |
| Cognitive ability | 0.131^**^ | 0.069 | 0.095^*^ |
|  | (0.044) | (0.044) | (0.048) |
| Postgraduate (ref. undergraduate) | -6.909^***^ | 2.427 | 6.062 |
|  | (1.514) | (1.864) | (3.405) |
| Part-time studies (ref. full-time studies | 9.141^***^ | 2.112 | -1.227 |
|  | (1.972) | (2.077) | (5.638) |
| Intermed. class * 24m | -0.005 | -0.037 | -0.065 |
|  | (0.063) | (0.045) | (0.052) |
| Intermed. class * 25-48m | -0.055 | -0.037 | 0.035 |
|  | (0.039) | (0.036) | (0.047) |
| Intermed. class * 49-72m | 0.043 | 0.077^*^ | 0.026 |
|  | (0.037) | (0.035) | (0.037) |
| Intermed. class * 73-96m | -0.021 | 0.007 | -0.037 |
|  | (0.040) | (0.043) | (0.03) |
| Intermed. class * 97-120m | -0.007 | -0.040 | -0.039 |
|  | (0.044) | (0.045) | (0.032) |
| Working class * 24m | -0.132^*^ | -0.085 | 0.022 |
|  | (0.052) | (0.059) | (0.074) |
| Working class * 25-48m | 0.023 | -0.000 | 0.023 |
|  | (0.078) | (0.053) | (0.049) |
| Working class * 49-72m | -0.016 | 0.099 | -0.013 |
|  | (0.058) | (0.058) | (0.036) |
| Working class * 73-96m | -0.003 | 0.074 | 0.016 |
|  | (0.073) | (0.053) | (0.036) |
| Working class * 97-120m | 0.063 | -0.010 | -0.080 |
|  | (0.067) | (0.049) | (0.072) |
| Intercept | 49.383^***^ | 50.226^***^ | 50.065^***^ |
|  | (1.415) | (1.048) | (1.451) |
| *Variance components* |  |  |  |
| Between-individual | 78.335 | 74.606 | 79.530 |
| Within-individual | 33.938 | 36.408 | 32.473 |
| Intraclass correlation (rho) | 0.70 | 0.67 | 0.71 |
| Chi^2^ | 84.03 | 101.96 | 90.06 |

*Source:* British Cohort Study 1970 (BCS70); *Note:* All models control for marital status, number of children and part-time employment. Robust standard errors are in parentheses. ^*^ *p* < 0.05, ^**^ *p* < 0.01, ^***^ *p* < 0.001.

FIGURE AIII. PREDICTED SIOPS BY PARENTAL CLASS


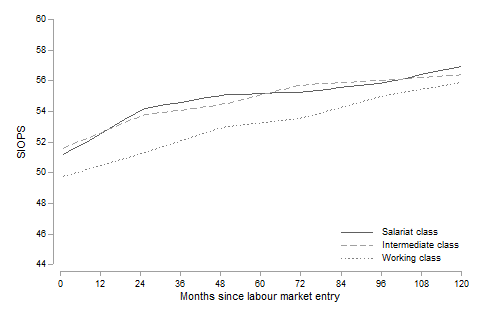


*Source:* British Cohort Study 1970 (BCS70); *Note:* Estimates obtained from growth curve model 5 in Appendix Table AVI.

FIGURE AIV. PREDICTED SIOPS BY PARENTAL CLASS ACROSS DIFFERENT FIELDS OF STUDY

*Source:* British Cohort Study 1970 (BCS70); *Note:* Respective estimates obtained from models 6-8 in Online Appendix Table AVII.
